# Problems in the Development of the Sleep–Wake Rhythm Influence Neurodevelopmental Disorders in Children

**DOI:** 10.3390/diagnostics13111859

**Published:** 2023-05-26

**Authors:** Kyoko Hoshino

**Affiliations:** Segawa Memorial Neurological Clinic for Children, Tokyo 101-0062, Japan; hoshino@segawa-clinic.jp

**Keywords:** development of sleep–wake rhythm, autism spectrum disorder, 3–4 months of age, sleep deprivation syndrome, circadian rhythm

## Abstract

Development of the sleep–wake rhythm has a significant effect on the physical and mental development of children. The sleep–wake rhythm is controlled by aminergic neurons in the brainstem’s ascending reticular activating system, which is associated with synaptogenesis and the promotion of brain development. The sleep–wake rhythm develops rapidly within the first year after birth. At 3–4 months of age, the framework of the circadian rhythm is established. The objective of the present review is to assess a hypothesis concerning problems in the development of the sleep–wake rhythm and their effect on neurodevelopmental disorders. Autism spectrum disorder is characterised by a delay in the development of sleep rhythms at 3–4 months of age and also insomnia and night-time awakenings, as supported by several reports. Melatonin may shorten the sleep latency in ASD. Rett syndrome sufferers kept awake during the daytime were analysed by the Sleep–wake Rhythm Investigation Support System (SWRISS) (IAC, Inc., (Tokyo, Japan)), and the cause was found to be the dysfunction of aminergic neurons. Children and adolescents with attention deficit hyperactivity disorder show sleep problems such as resistance to bedtime, difficulty falling asleep, sleep apnoea, and restless legs syndrome. Sleep deprivation syndrome in schoolchildren is deeply influenced by Internet use, games, and smartphones, and this syndrome affects emotion, learning, concentration, and executive functioning. Sleep disorders in adults are strongly considered to affect not only the physiological/autonomic nervous system but also neurocognitive/psychiatric symptoms. Even adults cannot avoid serious problems, much less children, and the impact of sleep problems is considerably greater in adults. Paediatricians and nurses should be aware of the significance, from birth, of sleep development and sleep hygiene education for carers and parents. This research was reviewed and approved by the ethical committee of the Segawa Memorial Neurological Clinic for Children (No. SMNCC23-02).

## 1. Introduction

The mammalian circadian clock is controlled by the oscillation of clock genes, located in the hypothalamic suprachiasmatic nucleus, and trained by light in the environment [1]. Most mammalian genes exhibit daily fluctuations, making circadian expression rhythms the largest known regulatory network in normal physiology [2]. Humans have lived on Earth as diurnal mammalians over many years. Insufficient sleep and/or sleep disorders in adults are tightly correlated to cardiovascular disease, diabetes, accidents and injuries, stress, and neurocognitive/psychiatric symptoms [3]. Development of the sleep–wake rhythm has significant influences on the physical and mental development of children. From birth, the sleep–wake rhythm changes on a daily basis. The baby grows day by day, with increasing waking time, feeding, and playing over the first year. 

The development of sleep–wake rhythms probably has a critical period, and is related to neurodevelopment [4]. By one year of age, most children eat three meals a day, sleep long hours at night, develop walking and language skills, and establish a diurnal rhythm as ‘humans living’ on the Earth. The nervous system and internal organs (digestive and endocrine systems) develop significantly in accordance with the sleep–wake rhythm [5]. Waking is controlled, not only by the aminergic neurons of the brainstem ascending reticular activating system, but also by orexin, histamine, and other neurotransmitters that are associated with synaptogenesis and promoting brain development. Arousal activates the autonomic nervous system and promotes the physical development of the respiratory, circulatory, and digestive systems [6]. How, then, are developmental and sleep disorders related? The causes of developmental disorders are diverse, and some are complicated by multi-organ diseases. However, the brainstem, which is one of the centres of sleep–wakefulness, has a broad influence on physical and mental development. I believe that the consolidation of the sleep–wake rhythm at the age of one year, which promotes the development of the brainstem, is the minimum requirement for normal physical and mental development in children. On the other hand, we considered that the different types of sleep disorders may indicate various neurodevelopmental disorders that correspond to sleep disorders. It may be possible to determine the point at which the abnormality begins to develop. We expect to find a significant relationship between sleep problems and neurodevelopmental disorders so far. In the present manuscript, I reviewed how the development of sleep and sleep problems influence the development of neurodevelopmental disorders. Moreover, we aimed to prove the hypothesis linking the development of the sleep–wake rhythm and neurodevelopmental disorders in this review based on our studies in sleep disorders and the pathophysiology of neurodevelopmental disorders so far. 

## 2. Normal Development of the Sleep–Wake Rhythm

### 2.1. Development of Sleep–Wake Rhythm

**Figure 1:**
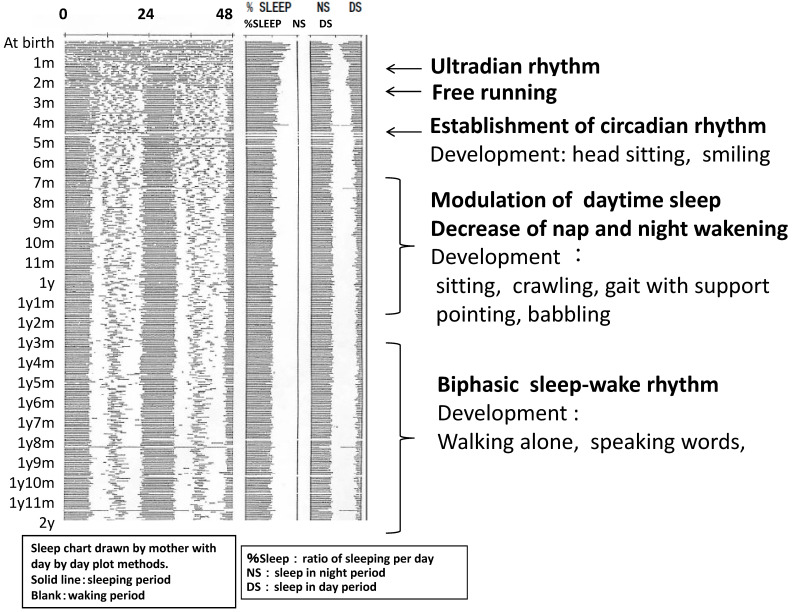
The development of sleep rhythms in a normal boy from the time of birth recorded by his mother with a day-by-day plot. Development of sleep (bold) and psychomotor development details are shown on the right. Circadian rhythms begin to distinguish between day and night by 3–4 months of age; head sitting and smiling to others become possible in normal development. Between 6–12 months of age, amplitude of sleep varies to decrease napping and night wakening. Psychomotor developments also vary. Around 12–18 months, the biphasic sleep–wake rhythm is largely established [8].

It is important to consider the question ‘over how many months in infancy does the baby establish the sleep–wake rhythm?’ However, it is difficult to answer this question because it is influenced by nutritional methods and weight gain. The development of sleep–wake rhythms has been investigated by several studies over the years [7]. Figure 1 [8] shows the development of sleep rhythms in a normal boy from the time of birth, and is frequently cited in Japan (Figure 1). The first few months of life are characterised by an ultradian rhythm in which the periods in the first few hours range in multiple levels of biological organization and adaptive significance in humans [9].

Sleep rhythms around two months of age become temporarily disordered as a free-running circadian rhythm; however, at around four months, babies gradually wake up in the morning, remain awake during the day, and sleep longer at night [10]. Children begin to distinguish between day and night by 3–4 months of age [10,11]. The transition between night-time and daytime sleep does not occur until approximately four months of age [5]. By six weeks of age, the child may sleep five to six hours continuously at night. This length increases to eight to nine hours at night by four months of age. Daytime sleep can be called ‘napping’. From six months to one year of age, daytime and night-time are determined more clearly, which means that the amplitudes of wakefulness and sleep are higher. The late infant begins to stay awake longer during the day and decreases the number of naps and awakenings during the night. By the age of one year, afternoon naps decrease and night-time awakenings almost disappear [12]. The basic structure of an infant’s circadian rhythms as a diurnal animal is established by around one year of age. 

In a cohort study of 194 cases, Hayama [13] found that infants with nocturnal rhythms at 4 months of age had disturbed the establishment of sleep–wake rhythms at 10 months of age. The pathophysiological mechanism of ‘night-time crying’ is unknown. Fukumizu [14] focused on the prevalence of night-time crying in healthy Japanese children and reported that 60% of them experienced night-time crying. Most children fall asleep again, which means they are self-soothing. Sleep problems in childhood may be caused by a dysfunction of the self-soothing mechanism. At 3–4 years of age, a sleep–wake rhythm was firmly established with a nap in the afternoon. The nap disappears between the ages of five and six and the circadian rhythm develops into that of adults.

In terms of sleep duration at night, sleep lengthens as development progresses. New-borns sleep 16–17 h, gradually decreasing to 14–16 h at four months of age and 13–14 h at six to eight months of age.

Night feeding decreases and daytime meals increase, according to development. The normal number of night-time feedings recorded by actigraphy was shown to decrease to one by one year of age [12]. The diurnal rhythms of the autonomic nervous system, such as body temperature, heart rate, and urination, are established during the first five months [15]. The earliest rhythm begins at approximately two to three weeks of age. Taken together, the physical development of children responds to the development of sleep–wake rhythms. The hypothesis asserting the relationship between irregular sleep rhythms and developmental disorders was researched in ASD [16,17].

### 2.2. Development of Rapid Eye Movement (REM) and Non-REM Sleep

There are two primary types of sleep: (1) active sleep or rapid eye movement (REM) sleep (dynamic REM sleep: eyes closed, eye movements, frequent movements of limbs and face, irregular breathing pattern) and (2) quiet sleep or non-REM sleep (static non-REM sleep: eyes closed without movements, little body movement, and regular breathing). Eye movements occur frequently at 24–26 weeks, with a period of no eye movement at 32 weeks, and regular breathing occurs at 32 weeks. Active sleep is primordial REM sleep, and is seen as a primitive, disorganized state during early embryonic development. It appears after 36 weeks of gestation and increases by three months [5]. In other words, the development of REM and non-REM sleep is the development of control mechanisms for excitation and inhibition and can be said to be the development of control systems for the brainstem and cortex. Thus, the development of REM and non-REM sleep is meaningful and reflects changes in brain maturation [18]. At one month of age, REM and non-REM sleep are frequently repeated, but by nine months of age, the sleep architecture approaches that of an adult. The twitching of the body during sleep may be due to muscle twitching associated with REM sleep. The development of REM and non-REM sleep is also linked to changes in the sleep–wake rhythm during infancy. The sleep–wake rhythm suggests alternating mechanisms of inhibition and excitation of the central nervous system during early development, which are closely related to the development of complex neural networks and neurotransmitters. Based on the above findings, we suggest that a well-regulated sleep rhythm is a well-developed control system for neurodevelopment in a well-developed child.

## 3. Developmental Abnormalities of the Sleep–Wake Rhythm

### 3.1. Autism Spectrum Disorder: Inability to Establish a Sleep–Wake Rhythm in Infancy

Reports on autism spectrum disorder (ASD) and sleep disorders in infancy and childhood are numerous. Nguyen [19] reported that sleep disturbances and night-time awakenings at one year of age correlate with ASD signs in two-year-olds. Verhoeff [20] analysed the association between sleep disorders in infants and ASD in a population-based cohort study. Lindor [21] found an association between the most common symptoms of ASD and sleep disorders. A recent Indian study by Samanta [22] found that 93% of carers reported sleep problems for ASD infants and 95% reported bedtime resistance. In recent years, it has been feared that night-time crying may increase the difficulty of fostering children with ASD, as it can elicit disturbances in attachment relationships [23].

We repeatedly emphasize the importance of the establishment of a sleep–wake rhythm at 3–4 months of age. Segawa [24] studied ‘the relationship between sleep rhythm and ASD in the first 3–4 months of life’ in 1979. We followed up on the results and proved that the dysregulation of sleep–wake rhythms at 3–4 months of age influenced the risk of ASD [25]. Fifty-two patients with neurodevelopmental disorders (including 18 with ASD) were examined retrospectively for (1) distinction between day and night at 3–4 months of age, (2) night-time crying, (3) sleep–wake rhythm by one year of age, and (4) sleep–wake rhythm in early infancy, based on patient medical records. Of the 34 children who ‘distinguished between day and night at 3–4 months of age’, 28 (82%) had ‘no night-time crying’, 30 (88%) had ‘good sleep–wake rhythm at 1 year of age’, and 22 (65%) had ‘good sleep rhythm in early infancy’, numbers which were significantly higher compared to those who had ‘no day or night discrimination’ (*p* < 0.05), as shown in Table 1.

**Figure 2:**
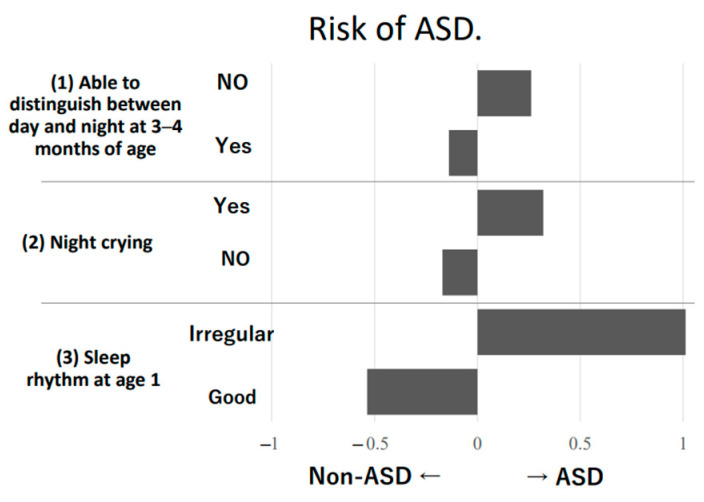
The accuracy and weight of each rating of sleep rhythm ((1), (2), and (3)) before the age of one year in predicting the subsequent diagnosis of ASD were examined. The statistical analysis on prediction used quantification theory II. Using quantification theory II to analyse the prediction equation with each rating of sleep rhythm ((1), (2), and (3)) up to age one as independent variables and the presence or absence of an ASD diagnosis as the dependent variable, not only the discriminant accuracy, but also the degree to which the judgment of each rating contributes to the prediction of diagnosis was determined. The higher the category score, the greater the contribution of each rating to prediction. These analyses were conducted using R (3.3.0). The discrimination accuracy of ASD diagnosis using category scores for each factor was 76.9%, and the contribution of each factor revealed that (3) “rhythm of sleep at age 1” had a significant impact on prediction. See Ref. [21].

Furthermore, ‘not distinguishing between day and night at 3–4 months of age’, ‘night-time crying’, and ‘poor sleep rhythm at the age of one’ were factors associated with the development of the risk of ASD (Figure 2). † Fisher’s exact probability test

I consider that an irregular sleep rhythm is not directly correlated to a cause–effect relationship but, rather, a relative relationship to ASD. The aetiology of ASD varies and it is not appropriate to assume that an irregular sleep rhythm solely causes it.

Contrastingly, excessive body movement during the REM period may cause awakenings, based on polysomnography studies of neurodevelopmental and involuntary movement disorders [26]. It has been reported that very low-dose levodopa therapy (VLDT) (0.5 mg/kg/day) can be effective in some cases. Hoshino et al. reported that VLDT reduced night-time crying and awakenings in patients with ASD or Rett syndrome and was suggested to ameliorate dopamine receptor hypersensitivity [27].

**Figure 3:**
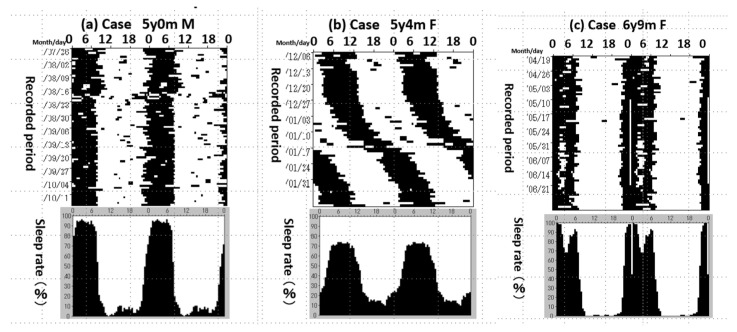
Sleep disorders in ASD. (**a**) He could not sleep easily and showed an irregular sleep rhythm. He kept awake in the middle of the night frequently. (**b**) She had severe ASD clinical features and suffered from a severe sleep disorder with free-running rhythm for one month. Her sleep rate was low in the middle of the night and high during the day. Her amplitude of the sleep–wake cycle was low. (**c**) She kept awake during midnight every night, even if she could sleep at a regular time. Her sleep rate at 2–3 am was decreased.

We analysed the sleep–wake rhythm using the Sleep–wake Rhythm Investigation Support System (SWRISS) (IAC, Inc.). The carer drew the sleep rhythm of the child and it was analysed with computer processing (Supplementary Figure S1). We analysed the sleep records of 66 ASD patients (mean age 6.4 ± 5.0 y, average of analysis period 3.9 ± 4.5 years). Fifty-three of the sixty-six patients (80.3%) presented with sleep disturbances [28]. Figure 3 shows the results of an analysis of three representative ASD children with severe sleep problems analysed with the SWRISS. All three cases showed severe sleep disorders with irregular sleep rhythms (a), free-running rhythms (b), and frequent waking in the middle of the night (c). The sleep rate of those cases was low in night-time and high in daytime. The amplitude of the sleep–wake cycle was decreased. Out of 53 patients, 64.1% were classified in (a), 3.4% in (b), and 18.9% in (c).

Children with ASD have been reported to have a delayed melatonin phase as part of the pathophysiology of ASD sleep disorders [29,30]. In Japan, long-term melatonin treatment for sleep problems in neurodevelopmental disorders (NDD) has been reported to improve not only sleep, but also behavioural problems [31]. Melatonin is available for treating neurodevelopmental disorders in Japan and its efficacy has already been reported [32]. The importance of adequate sleep hygiene interventions in the treatment of sleep disorders in ASD has been reported.

### 3.2. Rett Syndrome and the Inability to Keep Awake during the Daytime

**Figure 4:**
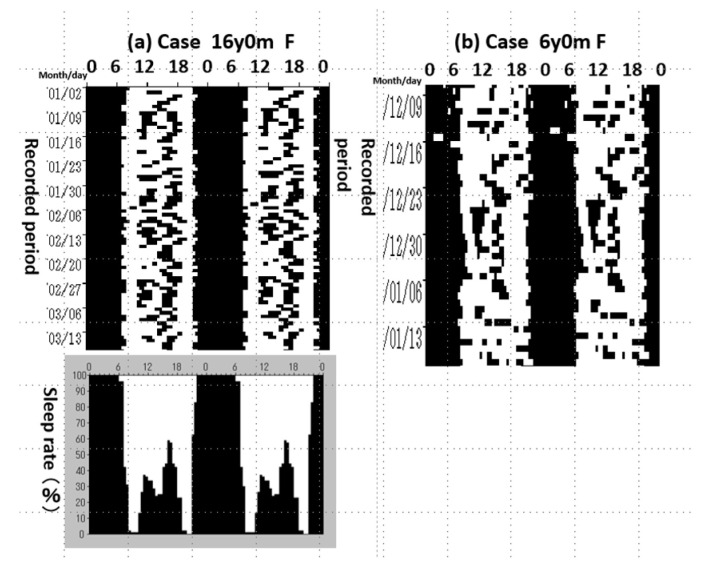
Day sleep in Rett syndrome. (**a**) Case 16y0m F and (**b**) Case 6y0m F.

From our retrospective study of the sleep rhythm in Rett syndrome [33], the subjects were characterised as ‘sleeping well at night but also during the day’, ‘quiet during the day’, and ‘not wanting milk unless we wake them up’, in contrast to ‘children who do not sleep at night’ and ‘children who sleep all the time’ in ASD. In the 2002 research project undertaken by Brain-Science & Education, of the Science and Technology Agency, Segawa also analyzed the sleep disorders of 13 patients with Rett syndrome [34] using the SWRISS, as shown in Figure 4.

Two subjects with Rett syndrome could not keep awake during the daytime, even though they slept enough at night. The aminergic neuron system activity in the brain stem was suggested to have decreased.

Nine patients (69.2%) appeared to have this type of sleep problem of sleeping during the daytime. Additionally, their carers and parents noticed hypotonia and poor feeding habits. The child neurologist diagnosed them with decreased muscle tone and truncal hypotonia during infancy. In both diseases, Segawa et al. explained them as abnormal nerve function in the brainstem. Rett syndrome has been reported to cause severe sleep disorders [35], insomnia, waking during the night, and frequent naps (microsleep during the daytime). We consider these sleep problems of Rett syndrome to suggest dysfunction of the aminergic neurons, but these problems differ from those of ASD.

### 3.3. Attention Deficit Hyperactivity Disorder: Sleep Problems

There have been many reports that 25–50% of children and adolescents with attention deficit hyperactivity disorder (ADHD) have trouble falling asleep, insomnia, waking during the night, and hypersomnia [36]. Konofal [37] reported that children with ADHD have resistance to bedtime, difficulty falling asleep, sleep apnoea, and restless legs syndrome (RLS). The study suggested that sleep quality is closely related to daytime sleepiness and concentration. Some experimental studies with adolescents diagnosed with ADHD have also reported this finding. Shorter sleep duration decreased performance on the continuous performance test (CPT), and sleep duration effected children’s inhibitory control, which was tested using the go/no-go task. When the children with ADHD got an extended night of sleep, inhibitory control improved by over 13% from baseline [36]. Nakatani [38] reported that children with ADHD predominantly exhibit more body movements during sleep, providing important insights into the pathophysiology of sleep problems in ADHD. As insomnia in children is a significant burden on parents, attention should also be paid to sleep disorders in children with ADHD. We also focus on narcolepsy or hypersomnia in this section because ADHD is highlighted in the comorbidities with hypersomnia/narcolepsy.

Typical narcolepsy is diagnosed definitively in school-aged children with cataplexy and low orexin levels in the spinal fluid. In recent years, idiopathic hypersomnia, which correlates with NDD, has been reported more frequently. It has been reported that 25% of patients with hypersomnia have ADHD, while 22% of patients with ADHD have hypersomnia [39]. Atomoxetine, methylphenidate, and other drugs may improve ADHD and hypersomnia. It has been reported that somnolence during the day, excess body movements during sleep, and a high apnoea–hypopnoea index are more common in patients with ADHD than in controls. For hypersomnia, a small dose of aripiprazole has been reported to be effective against shortened sleep duration and also exerts antidepressant effects [40].

Sleep apnoea is another factor that can be detrimental to sleep quality. Carter [41] conducted a study on children with Down syndrome and reported a high rate of daytime sleepiness combined with sleep-disordered breathing. Additionally, it was reported that sleep and behaviour problems improved after tonsillectomy in a child with ADHD [42].

### 3.4. RLS and Iron Metabolism

RLS also occurs in young children, who may cry, tap their feet, and ask their parents to massage them before sleeping. Some children who ‘resist going to bed’ complain that they cannot sleep because their legs are restless. Sometimes, patients with RLS cry loudly, which may be a serious problem for their carers. Iron and dopamine metabolism have been implicated in this disorder [43], and treatment is aimed at a serum ferritin level of at least 50 µg/mL. A single nightly dose of iron may dramatically improve the condition [44]. Iron has also been reported to be effective for NDD such as ASD, ADHD, and sleep [45]. Iron tablets are one of the drugs that are easy to use for general paediatricians.

### 3.5. Sleep Deprivation Syndrome: Emotion, Learning, and Concentration

Research carried out on preschool children with irregular sleep rhythms revealed problematic behaviours such as poor attention, impulsiveness, and anxiety [46]. From elementary school onwards, problems with sleep hygiene due to media, lessons, cramming for school, etc., can lead to chronic ‘sleep deprivation syndrome [47] and the emergence of abnormalities in emotion, learning, concentration, and executive functioning. In our clinic, since ‘sleep deprivation syndrome’ does not allow for a correct diagnosis of development and emotion, we first correct sleep and then make a diagnosis. It is important for the patient to understand the importance of sleep and to ‘try to go to bed early’ first, limiting media such as games. Medications should not be easily administered.

## 4. The Importance of Sleep Hygiene for Children

Before evaluating a sleep disorder in a child, it is of paramount importance to evaluate, as a prerequisite, whether ‘the parent or guardian has a stable sleep rhythm’ or whether ‘the parent or guardian pays attention to the adequate environment in which the child sleeps well at night’. The sleep rhythm of the child changes with that of his or her guardian [48]. A child with inappropriate sleep hygiene cannot be diagnosed as a ‘child who cannot sleep at night’ or as having a sleep disorder. It goes without saying that ‘sleeplessness’ is a ‘sleep disorder’, despite the efforts of the parents to put the child to sleep. Many studies suggest that the mother’s life influences the infant’s sleep rhythm.

Shinkoda [49] proved that the sleep time at four months of age was later than that at one and a half and three years of age, and that this was due to the mother’s sleep behaviour. Adachi [50] also found that the sleep rhythm at four months affects the rhythm establishment of the later sleep–wake rhythm and can be improved through intervention. Neurologists and paediatricians help prevent sleep deprivation by carers.

**Figure 5:**
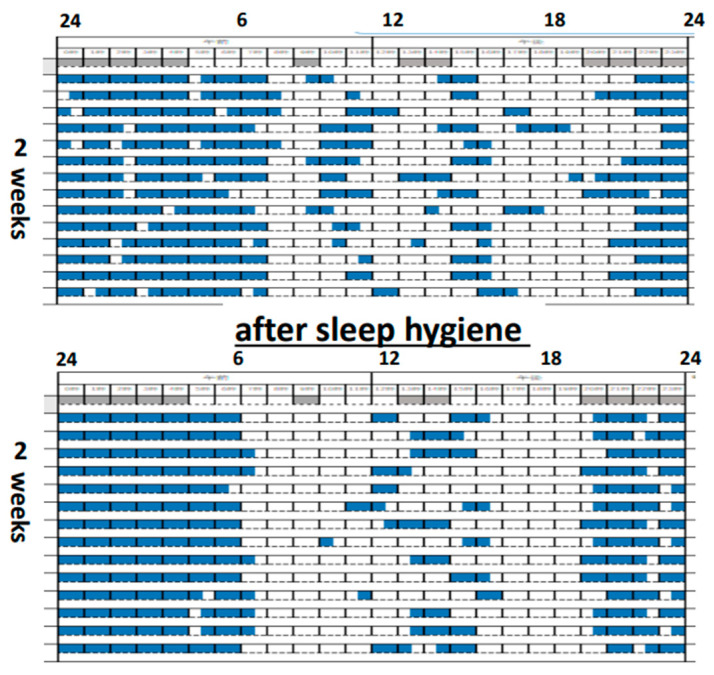
A 9-month-old boy improved not only his sleep rhythm, but his mood swings and developmental delays after two weeks of sleep hygiene education.

Figure 5 shows the sleep record of a nine-month-old boy. He visited our hospital with complaints of moodiness and developmental delay, chiefly. We instructed his parents to decrease feedings during the night, to wake him up in the morning (he used to wake up at 9 a.m.), and to communicate with him as much as possible at baby salons during the day. After two weeks, improvements in sleep rhythm, mood swings, and developmental delays were observed.

The disruption of sleep rhythms in early childhood can be a problem of inappropriate sleep hygiene and late sleep involving carers. Sleep problems in early childhood immediately affect children’s development. Suzuki [51] reported that irregular sleep rhythms in one-year-olds significantly correlated with poor finger-pointing, apathy, and emotional and behavioural problems. They also reported interesting findings on sleep and drawing triangles in five-year-old children. Late sleep time and the inability to draw triangles had a significantly higher odds ratio. The disintegration of higher cognitive function and sleep deprivation were also assessed. A five-year-old girl was presented to our hospital with the chief complaint of auditory hypersensitivity. Her bedtime was 11 p.m.; therefore, we instructed her to go to bed at 9 p.m. and her auditory hypersensitivity completely improved. This is the first case in which auditory sensitivity was improved by good sleep, and we are reminded of the importance of sleep in children.

It is no exaggeration to say that smartphones, the Internet, and the media have had the worst impact on children’s sleep in recent times. Children with ADHD are highly impulsive and game-dependent, whereas those with ASD are unable to leave the media due to interpersonal problems, such as developing social skills in the online world [52]. Night-time media use and light decrease melatonin levels, which further fails to improve sleep rhythms and contributes to abnormal sleep–wake rhythms. Media literacy is a major issue for children’s physical and mental development.

## 5. Conclusions and Future Directions

In summary, we believe that it is necessary to consider ‘child development’ through a focus on ‘sleep development’. Recently, a large survey of sleep–wake rhythms was conducted in Finland for one year [53]. It was reported that infants with slower sleep–wake rhythm development had a later sleep–wake rhythm, with more difficulties in settling to sleep; they woke frequently during the night. The researchers mentioned that the infants with the slow development of a sleep–wake rhythm showed short sleep periods and irregular circadian rhythms. They concluded that sleep quality and the quantity of infants’ sleep–wake cycles should be measured objectively and that interventions should be carried out to improve the sleep–wake rhythm and sleep quality in these infants. Subsequently to this study, there have been reports of studies of children’s sleep using new noninvasive devices [54] and the development of an interactive smartphone app with AI capabilities for the education of sleep hygiene [55]. Noninvasive new methods for in-home sleep measurements seem extremely convenient compared to full sleep studied in a laboratory that is equipped with an electroencephalogram, electromyogram, and a cardiorespiratory monitoring system. The aim of this new device is to precisely evaluate the sleep construction of developmental children, with there being less burden on not only the children but also parents [54]. The smartphone Nenne Navi app, the name of which means ‘sleep of baby’ in Japanese, supports reciprocal interactions between caregivers and paediatric sleep experts [55]. This device succeeded in improving night-time sleep duration and night-time awakenings (*p* < 0.001 for each). The researchers discussed how the feedback of users may improve children’s sleep habits using individualized advice sent to fit the families’ backgrounds and home lives.

We repeatedly emphasised the importance of establishing normal sleep–wake rhythm development early in infants. Paediatricians and nurses should be aware of the importance of sleep development and teach carers and parents proper sleep hygiene from birth, even during pregnancy. We suggest that a chart of sleep rhythms be included in the mother–child handbook for the understanding of carers and parents.

## Figures and Tables

**Table 1:** Of the 34 children who were able to distinguish between day and night at 3–4 months of age, 28 (82%) had “no night crying”, 30 (88%) had “regular sleep–wake rhythm at age 1”, and 22 had “regular sleep–wake rhythm in early childhood”. Those who were able to distinguish between day and night at 3–4 months of age had statistically significant less night-time crying, and regular sleep rhythms at 1 year of age and early childhood (Fisher’s exact probability test and residual analysis). See Ref. [25].

	Able to Distinguish between Day and Night at 3–4 Months of Age		
	YES (*n* = 34)	No (*n* = 18)	*p*
No night crying	28 (82%)	6 (33%)	0.001 †
regular sleep-wake rhythm at age 1	30 (88%)	4 (22%)	<0.001 †
regular sleep-wake rhythm in early childhood	22 (65%)	7 (39%)	0.015 †

## Data Availability

Data sharing is not applicable.

## Supplementary Materials

The following supporting information can be downloaded at https://www.mdpi.com/article/10.3390/diagnostics13111859/s1, Figure S1: Sleep–wake Rhythm Investigation Support System (IAC, Inc.).
