# Supplementary material for: Problems in the Development of the Sleep–Wake Rhythm Influence Neurodevelopmental Disorders in Children
Source: Diagnostics (Basel). 2023 May 26;13(11):1859. doi: 10.3390/diagnostics13111859 (PMC10252355; doi:10.3390/diagnostics13111859)
Supplement: Supplementary file 1 [file diagnostics-13-01859-s001.zip › diagnostics-2338137-supplementary materials.pdf]

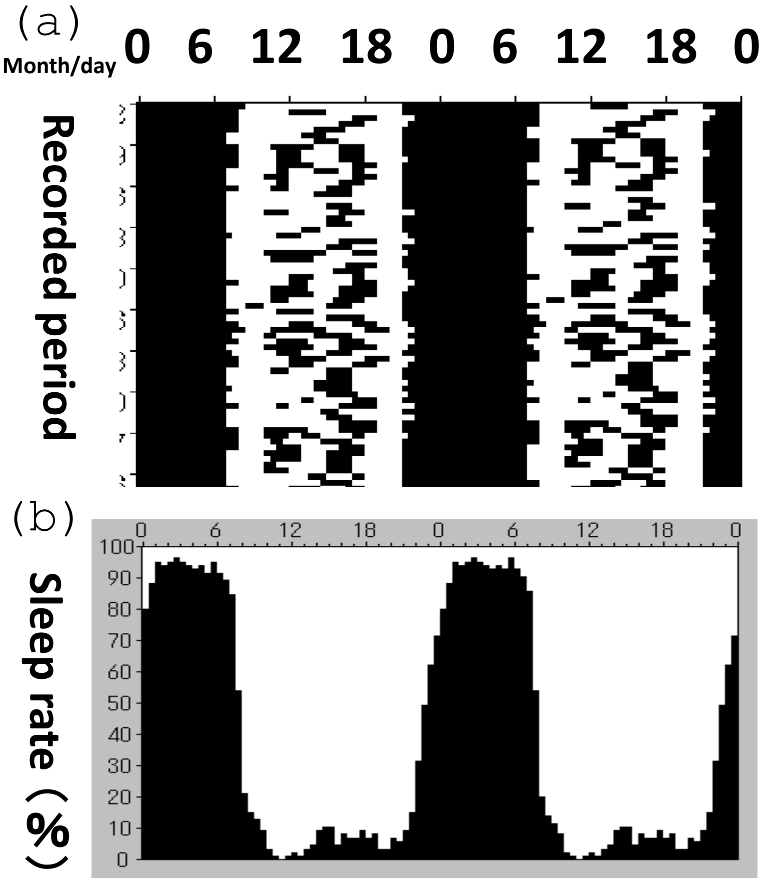

Figure S1. Sleep-Wake Rhythm Investigation Support System (IAC, Inc.)

(a) Caregiver draw a sleep rhythm of the child The results are analyzed by SWRISS. The horizontal axis represents the 48-hour day by day plot method. The vertical axis is the recorded period to be evaluated which is set by researchers .

(b) The sleep rate indicates the percentage of sleeping time corresponding time zone during the recorded period in the upper sleep chart. The sleep rate of this patients shows only 90% during midnight to morning.
